# Perioperative blood management programme reduces the use of allogenic blood transfusion in patients undergoing total hip and knee arthroplasty

**DOI:** 10.1186/s13018-016-0358-1

**Published:** 2016-02-29

**Authors:** Paul Kopanidis, Andrew Hardidge, Larry McNicol, Stanley Tay, Peter McCall, Laurence Weinberg

**Affiliations:** Department of Orthopaedic Surgery, Austin Hospital, Studley Road, Victoria, 3084 Australia; Department of Surgery, The University of Melbourne, Victoria, 3010 Australia; Department of Anaesthesia, Royal Darwin Hospital, Rocklands Drive, Tiwi, Northern Territory 0810 Australia; Department of Anaesthesia, Austin Hospital, Studley Road, Victoria, 3084 Australia; Anaesthesia Perioperative Pain Medicine Unit, University of Melbourne, Victoria, 3084 Australia

**Keywords:** Arthroplasty, Blood transfusion, Haemoglobin optimisation, Preoperative anaemia, Tranexamic acid

## Abstract

**Background:**

Optimisation of blood management in total hip (THA) and knee arthroplasty (TKA) is associated with improved patient outcomes. This study aimed to establish the effectiveness of a perioperative blood management programme in improving postoperative haemoglobin (Hb) and reducing the rate of allogenic blood transfusion.

**Methods:**

This retrospective before and after study involves 200 consecutive patients undergoing elective TKA and THA before (Usual Care group) and after (Intervention group) the introduction of a blood management programme in an Australian teaching hospital. Patients in the Intervention group underwent preoperative treatment for anaemia and received intraoperative tranexamic acid (15 mg/kg). The primary outcomes were to compare postoperative Hb levels and the rate of blood transfusion. Secondary outcomes included measurements of total amount of allogenic blood transfused, transfusion-related complications, postoperative complications, need for inpatient rehabilitation and duration of hospital stay.

**Results:**

There were no differences between baseline characteristics between groups. The mean (SD) preoperative Hb was higher in the Intervention group compared to that in the Usual Care group: 138.7 (13.9) vs. 133.4 (13.9) g/L, *p* = 0.008, respectively. The postoperative day 1 Hb, lowest postoperative Hb and discharge Hb were all higher in the Intervention group (*p* < 0.001). Blood transfusion requirements were lower in the Intervention group compared to the Usual Care group (6 vs. 20 %, *p* = 0.003). There were no differences in any of the secondary outcomes measured. Patients who were anaemic preoperatively and who underwent Hb optimisation had higher Hb levels postoperatively (odds ratio 5.7; 95 % CI 1.3 to 26.5; *p* = 0.024).

**Conclusions:**

The introduction of a perioperative blood optimisation programme improved postoperative Hb levels and reduced the rate of allogenic blood transfusion.

## Background

Surgery for total knee (TKA) and hip (THA) arthroplasty is common with a reported transfusion rate of approximately 26.8 % [1]. Eight to ten percent of all blood transfusions in the UK are due to TKA and THA surgery [2], and patients receiving blood from a matched donor carry a risk of blood transfusion-related complications [3, 4]. Furthermore, preoperative anaemia in elective TKA and THA is independently associated with transfusion and increased postoperative morbidity, supporting the need for preoperative evaluation and treatment [5].

The National Blood Authority in Australia has released a number of patient blood management initiatives recommending the establishment of a multidisciplinary, multimodal perioperative blood management programme to optimise preoperative red cell mass, minimise perioperative blood loss and tolerate postoperative anaemia [6]. Other blood management programmes are associated with improved patient outcomes and reduced length of stay [7]. In particular, it recommended that patients with preoperative anaemia are identified, and if iron deficient, preoperative iron therapy is advocated. [6]. Preoperative iron supplementation is suggested for orthopaedic procedures where there is a risk of severe postoperative anaemia to reduce the need for transfusion [8]. Approximately 20 % of patients who present for joint reconstructive surgery have coexisting anaemia, with 20 % of anaemic patients having anaemia responsive to iron therapy [9]. Intraoperative use of the anti-fibrinolytic tranexamic acid has also been shown to effectively reduce transfusion rates in TKR and THR without increasing the rate of other complications, including thromboembolic events [10, 11].

The aim of this study was to evaluate the effectiveness of the introduction of preoperative haemoglobin (Hb) optimisation programme together with intraoperative tranexamic acid to improve Hb levels postoperatively and reduce the rate of blood transfusion in patients undergoing primary elective THA and TKA.

## Methods

**Fig. 1:**
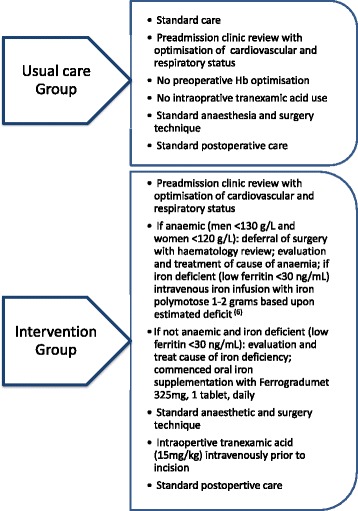
Overview of Usual Care and Intervention groups

Data from consecutive adult patients undergoing surgery for primary elective TKA and THA in a university teaching hospital were collected before (Usual Care group) and after (Intervention group) the introduction of a Hb optimisation programme that included the preoperative optimisation and treatment of anaemia (men <130 g/L and women <120 g/L) by a dedicated haematology clinic and the intraoperative use of tranexamic acid (15 mg/kg intravenously) (Fig. 1). This study is reported following the STROBE statement checklist for observational studies [12].

Standard perioperative care for both groups included strict transfusion practice in accordance with the Australian blood management perioperative guidelines [6]. In the absence of ischaemic heart disease, a transfusion trigger for blood transfusion was a Hb less than or equal to 80 g/L. Adjuncts to minimise intraoperative blood loss included the use of tourniquets in all total knee replacements. Haemostasis was achieved using diathermy without the use of adjunct fibrin sealants. Continuous passive motion and flexed knee approach in the first hour in TKA patients was not used. Standard postoperative care for all patients included a physical therapy-based early mobilisation programme, commencing on the day of surgery; multimodal analgesia led by a 24-h acute pain service; and enoxaparin 40 mg subcutaneous daily for venous thrombosis prophylaxis.

Exclusion criteria included patients with congenital or acquired coagulopathy, congenital or acquired thrombophilia, history of thromboembolism, allergy to tranexamic acid and revision surgery. Patients were identified using the Department of Orthopaedic audit database and the hospital’s health information system. The primary aims were to determine if patients in the blood management optimisation programme had higher Hb levels immediately postoperatively and on hospital discharge and if the rate of blood transfusion was lower compared to the Usual Care group. Secondary aims included measurements of total amount of allogenic blood transfused, transfusion-related complications, postoperative complications, need for inpatient rehabilitation and duration of hospitals stay.

Other data collected included patient characteristics including comorbidities, ASA status, preoperative incidence of anaemia, preoperative renal function, preoperative medications that impact on coagulation, surgical approach, duration of surgery, type of anaesthetic and amount of intravenous fluid administered. Postoperative complications were identified from patients’ admission histories, defined on the diagnoses written by the treating doctors in patients’ progress notes, with cross-checking with laboratory and radiological data. The data was collected by a principal investigator (PK) and with the assistance of a research nurse.

At our institution, the proportion of patients prior to the study that required a blood transfusion in the general orthopaedic setting was 25 %. A sample size calculation of 194 subjects was based upon an 80 % chance of detecting, as significant at the 5 % level, a decrease in the primary outcome measure from 25 % transfusion rate in the Usual Care group to 10 % in the Intervention group. To allow for dropout and missing data, we collected information from 200 patients. Results were statistically analysed using GraphPad (Prism 6^®^, Version 6.0b) with independent parametric data analysed with unpaired *t* tests, non-parametric nominal data using chi-square test and Mann-Whitney *U* test for independent ordinal data. Backward multivariate regression modelling was undertaken using SPSS (IBM SPSS Statistics for Macintosh, Version 22.0) to examine for independent factors associated with higher Hb levels in the postoperative period and on hospital discharge. A *p* value of <0.05 was considered statistically significant. The Austin Health Research Ethics Committee granted prospective ethics approval (HREC no H2013/04904).

## Results

**Fig. 2:**
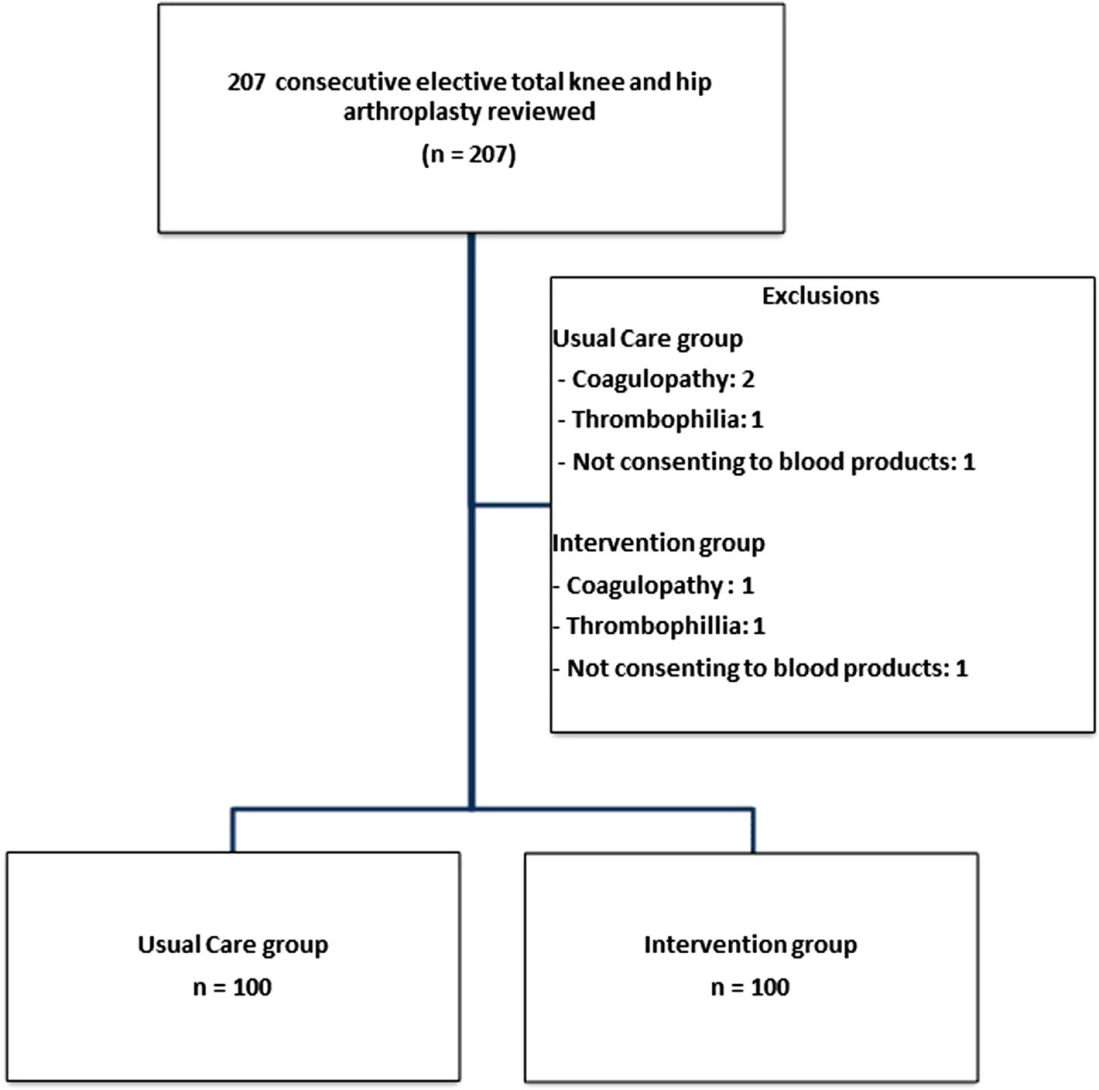
Overview of total number of patients

Data from 200 consecutive patients were collected between 2011 and 2013. Figure 2 outlines the numbers of patients who were included and excluded in the study. There were no differences between groups for baseline patient characteristics, ASA status, comorbidities, or preoperative medications relevant to bleeding (Table 1).

**Table 1:** Baseline patient characteristics. Data presented as mean (standard deviation) or number and absolute percentages

	Usual Care group	Intervention group	*p*
	*N* = 100	*N* = 100	
Sex; M:F	26:74	32:68	0.350
Age; years (SD, range)	68.1 (9.7, 46–94)	67.2 (10.9, 13–88)	0.547
Weight; kg (SD, range)	81.6 (17.8, 46–130)	83 (18.5, 50–137)	0.566
Body mass index; kg m^−2^	31.7 (6.4)	31.4 (6.7)	0.757
American Society Anesthesiology Status			
1	4	3	
2	56	73	0.971
3	39	23	
4	1	1	
Comorbidities			
Cardiac disease	18	17	0.852
Hypertension	65	62	0.660
Diabetes mellitus	22	16	0.280
Peripheral vascular disease	4	2	0.407
Cerebrovascular disease	6	1	0.054
Pulmonary embolus	1	0	0.316
Deep venous thrombosis	4	7	0.352
Renal impairment	5	11	0.118
Active smoker	10	5	0.180
Previous smoker	33	39	0.377
Chronic obstructive airways disease	20	21	0.861
Other	71	78	0.256
Preoperative medications			
Aspirin	17	17	1.000
Clopidogrel	1	0	0.316
Warfarin	0	0	1.000
Non steroidal anti-inflammatory drugs	28	19	0.133

The preoperative biochemistry and haematological results are presented in Table 2. There was no difference in coagulation profiles or renal function markers between groups. In terms of operative data, there was no difference between groups in the type of surgery, surgical approaches or total number of surgeons performing the operation. There was no significant difference in the number of patients who received a surgical drain (Usual Care group 6 % vs. Intervention group 4 %). The type of anaesthesia delivered did not differ except for a reduction in femoral nerve blocks inserted in the Intervention group. Intraoperatively, the mean (SD) fluid intervention was 1862 (609) mL vs. 2072 (736) mL in the Usual Care group, *p* = 0.029. However, at 24 h, fluid intervention did not differ significantly. The median (interquartile range (IQR)) duration of surgery was 120 (90:135) min in the Intervention group vs. 120 (105:150) min in the Control group, *p* = 0.0254.

**Table 2:** Summary of preoperative biochemistry and haematology; data are presented as mean and standard deviation

Preoperative	Usual Care group	Intervention group	*p*
	*N* = 100	*N* = 100	
Haemoglobin (g/L)	133.4 (13.9)	138.7 (13.9)	0.008
Mean corpuscular volume (fL)	89.9 (7.3)	88 (4.7)	0.032
Creatinine (μmol/L)	74.3 (20.4)	75 (19.5)	0.809
Estimated glomerular filtration rate (mL/min/1.73 m^2^)	79.8 (57.7)	76.8 (14.2)	0.621
Albumin (g/L)	41.1 (4.3)	40 (3.1)	0.036
Prothrombin time (s)	11.2 (1.8)	11 (1)	0.501
Activated partial thromboplastin time (s)	27.2 (4.7)	26.8 (4.1)	0.459

**Fig. 3:**
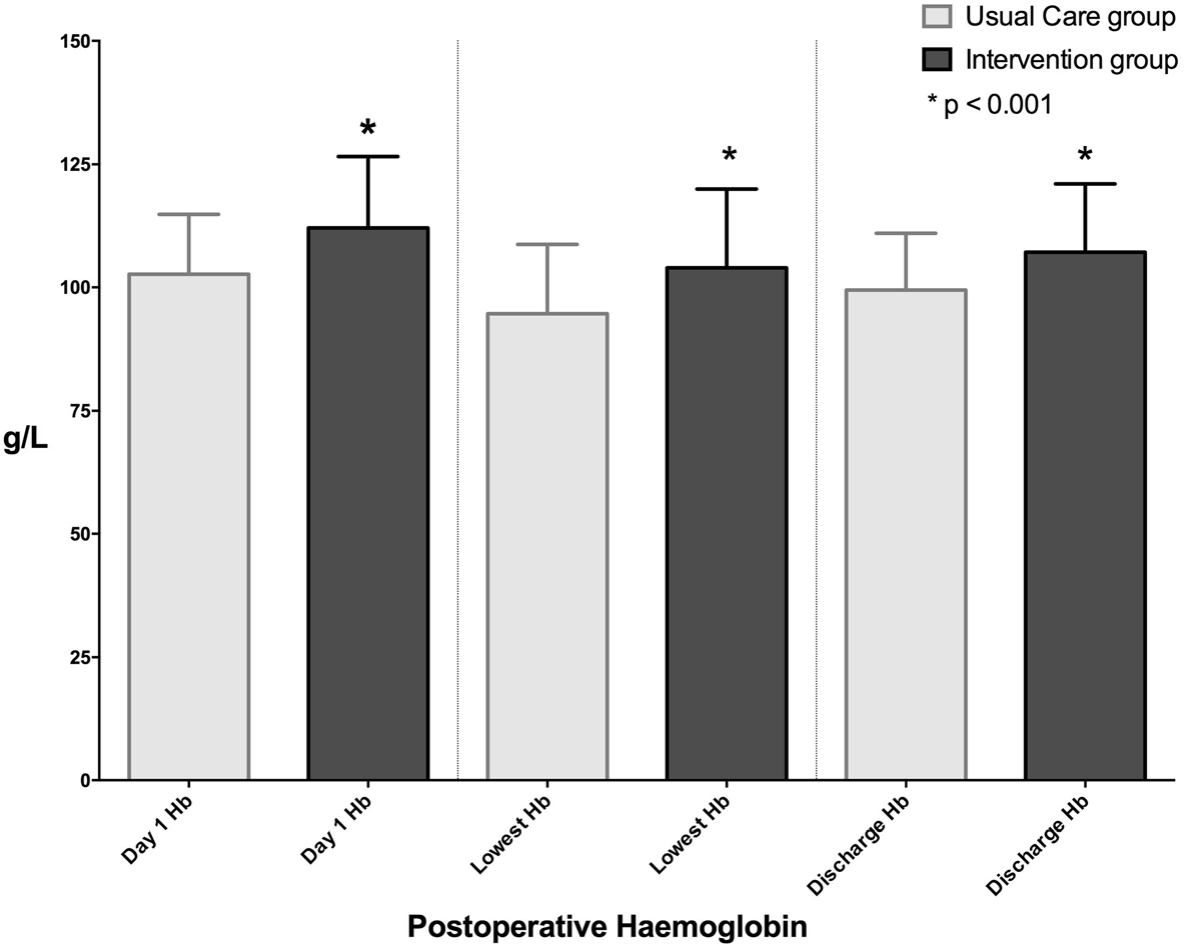
Postoperative haemoglobin values expressed as mean and standard deviation

In the Usual Care group, iron studies were not part of standard of care and no patient received preoperative anaemia optimisation. Eighteen patients (18 %) in the Usual Care group were anaemic preoperatively (lowest Hb 88 g/L). Of the anaemic patients in the Usual Care group, 9 (50 %) required a perioperative blood transfusion. In the Intervention group during screening preoperatively, 18 patients (18 %) were anaemic or had low ferritin levels (<30 ng/mL) (lowest Hb 100 g/L). Seven patients required iron supplementation, five patients required dietary or cessation of alcohol advice and six patients were optimised for other causes of anaemia including anaemia of chronic disease. No patients received erythropoietin. After the Hb optimisation programme, seven patients (7 %) remained anaemic preoperatively (lowest Hb 100 g/L), with one of these patients requiring a perioperative blood transfusion. Day 1 Hb, lowest postoperative Hb and discharge Hb were all significantly higher in the Intervention group (*p* < 0.001). These are summarised graphically in Fig. 3.

Twenty patients (20 %) in the Usual Care group required a perioperative blood transfusion vs. six patients (6 %) in the Intervention group (*p* = 0.003). The median (IQR) number of units of blood transfused was 2 (1:2.75) units in the Usual Care group and 2 (2:4.75) units in the Intervention group (*p* = 0.126). The median (IQR) Hb transfusion trigger was 76.0 (71: 82) g/L in the Usual Care group vs. 74.0 (IQR 67: 87) g/L in the Intervention group (*p* = 0.98). There were no differences between groups in the length of hospital stay, need for inpatient rehabilitation, complication rates or mortality (Table 3).

**Table 3:** Patient outcomes and complication rates. Data expressed as median (interquartile range) or absolute number or percentage

	Usual Care group	Intervention group	*p*
	*N* = 100	*N* = 100	
Length of stay (h)	101.3 (98:128)	102.0 (84:125)	0.892
Discharge destination			
Home	79	87	0.187
Inpatient rehabilitation	21	13	
Complication rate			
Total number of complications	16	17	0.849
Major complications	5	7	0.552
Acute coronary syndrome	3	0	0.081
Exacerbation of chronic lung disease	0	1	0.316
Exacerbation of heart failure	0	1	0.316
Acute kidney injury	1	2	0.561
Pulmonary embolism	0	0	1.000
Narcotised from analgesia	1	3	0.312
Minor complication	11	10	0.818
Hypotension	8	5	0.390
Febrile non-haemolytic transfusion reaction	1	2	0.561
Adverse drug reaction	1	0	0.316
Delirium	1	0	0.316
Deep vein thrombosis	0	0	1.000
Acute urinary retention	0	1	0.316
Uretheral injury	0	1	0.316
Clostridium difficile colitis	0	1	0.316
Patient outcomes			
Medical emergency team response	9	8	0.800
• Hypotension	7	5	0.390
• Oliguria	1	0	0.316
• Low respiratory rate	1	3	0.312
Cardiorespiratory arrest	0	0	1.000
Mortality	0	0	1.000
Intensive care admission	1	2	0.561
Readmission	2	0	0.155
• Wound cellulitis	1	0	0.316
• Hip dislocation	1	0	0.316

Preoperative anaemia alone was strongly associated with the requirement for a perioperative blood transfusion (odds ratio 6.1; 95 % CI 3.0 to 12.2). On multivariate regression modelling, patients who were anaemic preoperatively and who underwent perioperative Hb optimisation (i.e. preoperative treatment for anaemia plus intraoperative tranexamic acid) had higher Hb levels immediately postoperatively (odds ratio 5.7; 95 % CI 1.3 to 26.5; *p* = 0.024). A drop in Hb by 30 g/L or more on postoperative day 1 (odds ratio 0.19; 95 % CI 0.05 to 0.99; *p* = 0.048) and age greater than 65 years (odds ratio 0.19; 95 % CI 0.04 to 0.86; *p* = 0.031) were the only factors associated with Hb less than 100 g/L on hospital discharge. Use of either tranexamic acid alone or iron optimisation alone, ASA status, duration of surgery, individual surgeons, intraoperative fluid use, gender and BMI > 30 kg/m^2^ were not associated with lower transfusion rates or higher Hb values in the postoperative period.

## Discussion

### Key study findings

We performed a retrospective quality assurance programme evaluating the effectiveness of a change of practice of preoperative treatment of anaemia utilising iron supplementation together with intraoperative tranexamic acid as recommended by National Blood Authority Patient Blood Management Guidelines [6] to reduce the rate of allogenic blood transfusion at our institution. We found that the introduction of the blood optimisation programme reduced the incidence of preoperative anaemia. In addition, patients undergoing elective THA and TKA who underwent a perioperative blood management programme had a lower incidence of allogenic blood transfusion. Patients who were anaemic preoperatively benefited most from the programme. This has significant pharmacoeconomic and resource considerations for institutions implementing similar blood management strategies.

### Relationship to previous studies

Previous studies have aimed to define an optimal strategy for blood conservation in the setting of major orthopaedic joint surgery. Kotze et al. performed a retrospective study of 717 TKA and THA in the UK, analysing the effect of instituting a blood management programme that included the use of preoperative iron transfusions with erythropoietin and intraoperative tranexamic acid with cell salvage devices [2]. The programme significantly reduced the rate of allogenic blood transfusion and length of stay. In our study, we observed that there was no difference to length of hospital stay between patients in the blood management programme and the Usual Care groups. Similar findings were reported by Wong et al. [13], who evaluated the effectiveness of a comprehensive blood conservation algorithm using a cluster randomisation trial design involving 29 hospitals. Their programme consisted of specific blood conservation strategies including recombinant human erythropoietin or autologous blood donation and transfusion guidelines. Similar to our study, they demonstrated that the comprehensive blood conservation was superior to usual care for reducing allogenic transfusion, despite no differences to length of hospital stay.

Our programme utilised treatment of preoperative anaemia including use of iron supplementation and intraoperative tranexamic acid. Previous studies in orthopaedic procedures have demonstrated that preoperative iron supplementation can reduce the need for transfusion, particularly in the context of expected postoperative anaemia [8]. Approximately 20 % of patients who are anaemic at presentation for joint reconstructive surgery will have an anaemia that is responsive to iron therapy [8, 9]. On regression modelling, we observed that patients who appeared to benefit most from the programme were the patients who were anaemic preoperatively and who underwent the blood management programme. In the patients with preoperative anaemia who are also iron deficient, iron loss due to surgical bleeding can exaggerate a preexisting iron deficiency and may worsen postoperative anaemia. Our findings of a drop in Hb by 30 g/L or more on postoperative day 1 and age greater than 65 years were negative, associated with Hb > 100 g/L on discharge; are also physiologically plausible, i.e. the greater the intraoperative blood loss, the lower the discharge Hb; and impaired erythropoiesis in older patients [14].

Data from meta-analyses have previously supported tranexamic acid in blood conservation in THA and TKA without predisposing to thromboembolic complications [10, 11]. In our study, similar to these studies, we observed no complications relating to use of tranexamic acid. Interestingly, we found that the use of tranexamic acid was not an independent factor associated with improvements of Hb postoperatively. However, our study aims were to evaluate the effectiveness of the introduction of preoperative Hb optimisation programme together with intraoperative tranexamic acid to improve Hb levels postoperatively and reduce the rate of blood transfusion. Inferences should therefore not be made about the independent effectiveness of tranexamic acid alone, as the study was not designed nor powered for this purpose. The dosing strategy used in our study (15 mg/kg) is considered a ‘low’ dose regime [10, 11], and further research is still required to determine the optimal dosing of tranexamic acid in the setting of major arthroplasty surgery.

In addition to our study, we noted a significant reduction in the operative time in the Intervention group. Reductions in operative times related to intraoperative tranexamic acid use was not reported in three large meta-analyses examining the use of tranexamic acid in patients undergoing total hip and knee arthroplasty [10, 11, 15]. It is plausible that the use of tranexamic acid reduced time to achieve haemostasis, hence reducing the duration of surgery. However, we consider an effect size of 12 min for total duration of surgery to be of little clinical significance. Future studies will need to validate this finding with more robust study design for this particular endpoint with consideration of its clinical and economic importance.

### Study implications

The clinical implications of our findings suggest that a blood optimisation programme can safely and significantly reduce the rate of allogenic blood transfusion in accordance with recommendations by National Blood Authority Patient Blood Management Guidelines [6]. In addition, our findings provide support for the practice change of deferral of surgery for investigation and treatment of anaemia, with the combined used of tranexamic acid for blood conservation. Importantly, as evident by our findings, the use of tranexamic acid or iron optimisation alone were not associated with lower blood transfusion requirements or higher Hb levels postoperatively, reinforcing the importance of multimodal blood management strategies rather than single interventions. Future studies could explore whether these measures provide any pharmacoeconomic benefits to such an intervention.

### Strengths and limitations

There are several limitations to this quality assurance retrospective before and after study. Firstly, the study design does not establish a causal relationship of the Hb optimisation programme and the reduction in blood transfusion or higher postoperative Hb levels observed between the two groups. These associations may be subject to bias from selection, confounding or random error, although we attempted to control for confounders by using regression analysis. Specific biases regarding surgical decisions during the operation or the need for blood transfusion cannot be excluded. However, the surgical technique, surgeon and Hb transfusion triggers, amongst the other variables outlined above, appeared to be similar in both groups. Our findings only apply to this type of surgery; therefore, they should not be generalised to other orthopaedic or surgical procedures. Furthermore, the external validity or generalisability of our results to other hospitals is limited, since we collected data only from a single institution. Although we report a low incidence of deep vein thrombosis, which was not different between the groups, we did not use ultrasound to identify this complication, as in our institution this non-invasive imaging tool has been shown to have a low sensitivity for detecting deep vein thrombosis. Importantly, our institution does not routinely record blood loss in the drains and was unable to accurately quantify total blood loss. The dose of tranexamic acid used in our programme may be different to other centres, and this may warrant further evaluation. We did not perform any pharmacoeconomic evaluation of the blood optimisation programme; however, this should not detract from the study aims or objectives. Finally, the intervention programme included a combination of both preoperative haemoglobin optimisation and tranexamic acid; the utility of the study is therefore limited in drawing conclusions on each separate component of the programme as eluded above.

## Conclusions

The introduction of a preoperative blood optimisation programme utilising iron supplementation combined with intraoperative tranexamic acid has effectively reduced the use of allogenic blood transfusion in elective total hip and knee arthroplasty surgeries at our institution. Patients who were anaemic preoperatively benefited most from the programme. Our findings support the use of a multidisciplinary blood optimisation programme in this setting. These results can be used to build hypotheses for further controlled trials.

### Ethics approval

This study received prospective Austin Health Research Ethics Committee approval (HREC no H2013/04904).
